# LLM-powered TNM staging of neuroendocrine tumors from PET/CT reports

**DOI:** 10.1186/s12880-025-02092-3

**Published:** 2025-12-23

**Authors:** Markus Mergen, Daniel Spitzl, Matthias Eiber, Rickmer F. Braren, Lisa Steinhelfer

**Affiliations:** 1 Institute for Diagnostic and Interventional Radiology, School of Medicine and Health, TUM Klinikum, Klinikum rechts der Isar, Technical University of Munich (TUM), Ismaningerstr. 22, 81675 Munich, Germany; 2 Department of Nuclear Medicine, School of Medicine and Health, TUM University Hospital, Technical University Munich, Munich, Germany; 3 German Cancer Consortium (DKTK), Partner-Site Munich, DKFZ and Klinikum rechts der Isar, Munich, Germany; 4 Bavarian Cancer Research Center (BZKF), Munich, Germany

**Keywords:** Large language models, Neuroendocrine tumors, TNM staging, PET/CT, Clinical decision support

## Abstract

**Purpose:**

Imaging reports are essential for the diagnostic evaluation, treatment planning, and follow-up of patients with neuroendocrine tumors (NETs) of the gastroenteropancreatic (GEP) system. The tumor-node metastasis (TNM) classification is a common model for evaluating the prognostic value of tumor patients. However, their traditional free-text format varies in structure, detail, and clarity, leading to inconsistencies and potential omissions of critical information necessary for optimal patient management. Recent advancements in large language models (LLMs) have created new opportunities for automating complex medical assessments, including the extraction of UICC and ENETS staging classifications from imaging reports. This approach aims to improve standardization, enhance clarity, and ensure consistency, ultimately facilitating more effective multidisciplinary clinical decision-making. This study evaluates whether large language models (LLMs) can infer UICC and ENETS TNM stage for GEP‑NETs from PET/CT free‑text reports that contain descriptive findings only (no explicit TNM labels).

**Methods:**

We evaluated several models, including ChatGPT-4o, DeepSeek V3, Claude 3.5 Sonnet, and Gemini 2.0 Flash, on a physician-generated fictitious dataset of 108 PET/CT reports with expert-annotated TNM classifications according to UICC and ENETS criteria. Model performance was assessed through F1-scores, comparing LLM-generated classifications against human expert benchmarks.

**Results:**

Among the tested models, ChatGPT-4o demonstrated the highest accuracy, achieving microF1 scores of 0.79, 0.99 and 0.99, for T, N and M according to UICC and 0.84, 1.00 and 0.99 respectively, according to ENETS. These results indicate that LLMs have the potential to assist in oncologic staging of NETs, especially offering support for non-specialists in clinical decision-making. However, before integration into routine practice, further prospective validation and rigorous evaluation in real-world settings are necessary.

**Conclusion:**

This study underscores the promise of LLMs in oncologic workflows while highlighting the importance of robust benchmarking and clinical validation.

**Supplementary Information:**

The online version contains supplementary material available at 10.1186/s12880-025-02092-3.

## Introduction

The integration of artificial intelligence (AI) into medical workflows is transforming the landscape of clinical decision-making [1, 2]. Large language models (LLMs) have shown remarkable capabilities in processing complex medical text, raising the possibility of their use in oncologic classification [3]. Accurate tumor staging is critical for determining prognosis and guiding treatment decisions, yet, it remains a highly specialized task that demands expert interpretation [4]. Given the increasing volume of medical data, AI-driven approaches have the potential to enhance efficiency and reduce variability in staging assessments [5–7]. However, ensuring that LLMs can reliably interpret and apply structured classification systems, remains a key challenge [8]. While LLMs have demonstrated strong performance in tasks such as clinical summarization and information extraction, their ability to classify disease extent according to structured staging criteria remains largely untested [9, 10]. Gastroenteropancreatic neuroendocrine tumors (GEP-NETs) are a heterogeneous group of neoplasms with highly variable clinical behavior, ranging from small, well-differentiated lesions amenable to curative endoscopic resection to aggressive, poorly differentiated malignancies with diffuse metastases and poor survival outcomes [11, 12]. Their classification has historically been challenging and inconsistent since their initial description in 1907 [13]. PET/CT is integral to the management of GEP-NETs, supporting initial staging and restaging, guiding therapy selection (including peptide receptor radionuclide therapy), and monitoring treatment response through whole-body assessment of somatostatin receptor expression. GEP-NETs are staged using two established frameworks: the UICC TNM and the ENETS classifications. While both define stage via tumor extent, nodal involvement, and distant metastasis, ENETS incorporates site-specific criteria that can diverge from UICC; both systems are used in clinical practice, so we evaluate model performance against each. Accurate tumor classification is crucial, as even minor discrepancies in interpretation can substantially influence staging, therapeutic decisions, and overall patient management. In current clinical practice, however, such information is typically embedded within unstructured free-text reports, requiring manual extraction to obtain standardized classifications.

Artificial intelligence (AI) approaches—particularly those leveraging large language models (LLMs)—offer the potential to automate this process and improve the consistency of oncologic staging [14]. Despite rapid advances in AI for medical imaging, most prior research has centered on image-based deep learning, whereas the application of LLMs to text-based oncologic classification remains largely unexplored [15]. 

## Materials and methods

This retrospective study was conducted under ethics approval 2024-590-S-CB from institutional review board of the Technical University of Munich, with all methods carried out in compliance with relevant guidelines and regulations. The requirement for individual informed consent was waived by the ethics committee due to the retrospective design and the use of fully anonymized clinical data.

This study examined 108 fictitious PET/CT reports with respective TNM assignmnets, according to ENET [16–20] and UICC [21] of neuroendocrine tumors generated by two nuclear medicine physicians. As only fictitious data was used, the need to obtain informed consent was waived by the institutional review board of the Technical University of Munich. The reports were systematically created to closely resemble real-world clinical reports in terms of structure, terminology, and level of detail. To ensure consistency and standardization, a predefined template was used to guide the generation of reports. Explicit TNM labels were not included in these reports. The template was designed based on established radiological reporting standards and adapted to reflect common linguistic patterns observed in real clinical documentation. Because all reports were prospectively generated for this study, no clinical time period applies; all 108 template‑conforming reports were included. The case distribution reflects a heterogeneous cohort, representative of a balanced representation of different GEP-NETs. Each report was independently reviewed by at least one additional radiologist to ensure accuracy and adherence to the standardization criteria. Discrepancies in descriptions or classifications were resolved through consensus discussions among the three medical experts. Ambiguous phrasing was included to simulate real-world variability. An example PET/CT report with corresponding ground-truth TNM classification and model-generated outputs can be seen in Supplementary Table 1.

Four models were evaluated in a zero-shot setting:

- ChatGPT-4o (May 2024 version, gpt-4o-2024-05-13)
- DeepSeek V3
- Claude 3.5 Sonnet (claude-3-5-sonnet-20240620)
- Gemini 2.0 Flash (gemini-2.0 flash experimental)

All four models were tested in a zero-shot setting using the same standardized prompt template verbatim:

Accurately TNM classify the following imaging report of a neuroendocrine neoplasia after the newest version of UICC TNM classification Staging System

and

Accurately TNM classify the following imaging report of a neuroendocrine neoplasia after the newest version of the European Neuroendocrine Tumor Society (ENETS) Staging System.

The dataset size was selected to ensure a well-balanced representation of various GEP-NETs while maintaining practical feasibility. Including 108 NETs enabled a meaningful assessment of classification performance. To reduce potential bias towards more common NET localizations, efforts were made to achieve a representative distribution.

Performance was computed separately for T, N, and M for each system. Accuracy was defined as the proportion of cases where the exact TNM classification was correctly predicted compared to the ground truth. The Python packages NumPy (version 1.26.4), pandas (version 2.2.0), scikit-learn (version 1.4.0), statsmodels (version 0.14.1), matplotlib (version 3.8.2), and seaborn (version 0.13.2) were used for data analysis and visualization [22–26]. Each of the TNM components (T, N, and M) was evaluated as a multi-class classification task. Metrics were computed using a one-vs-rest approach for each category, meaning that misclassification from one class to another contributed to both a false positive and a false negative across the respective categories. Micro-averaging was applied to combine counts across all classes, while macro-averaging reflected the unweighted mean of class-specific results.

Precision was defined as the proportion of correctly predicted cases among all cases predicted for a given class, while recall represented the proportion of correctly predicted cases among all true cases for that class. The F1 score was computed as the harmonic mean of precision and recall. Macro-F1 was obtained as the unweighted average of F1 scores across all classes, giving each category equal importance, whereas Micro-F1 was derived from the pooled counts of true positives, false positives, and false negatives across all classes, thereby weighting the results according to class frequency.

## Results

**Fig. 1:**
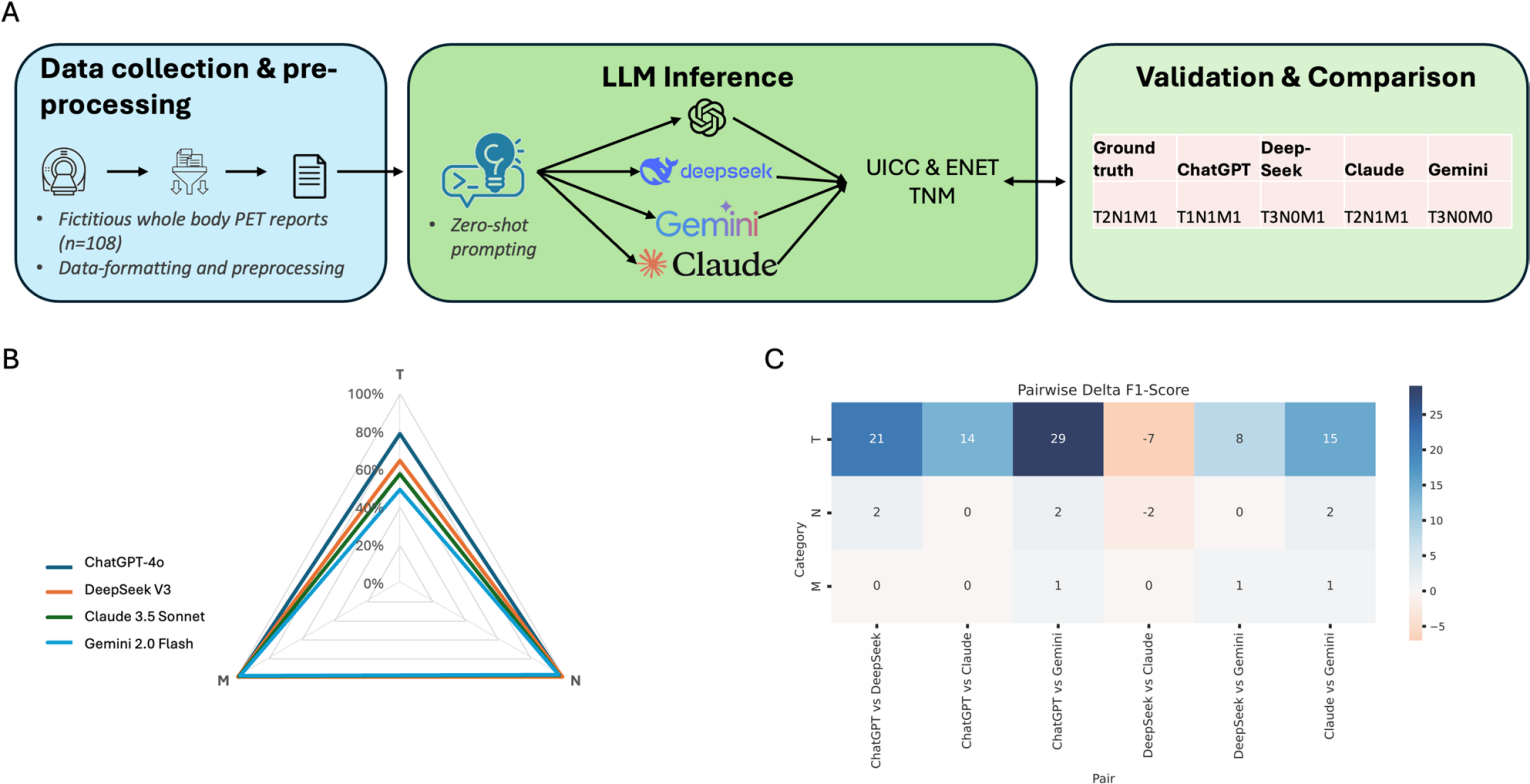
(**A**) Schematic representation of the workflow for classifying NET UICC and ENETS TNM based on PET/CT reports. The process includes PET/CT report generation, LLM-based classification, and validation against ground truth. (**B**) Radar chart illustrating the hit rate for UICC TNM classification by ChatGPT-4o, DeepSeek V3, Claude 3.5 Sonnet, and Gemini 2.0 Flash. Key findings include ChatGPT-4o´s high F1 scores (0.79) for T classification. (**C**) shows a heatmap depicting the differences in performance for classifying T, N and M stage across models used

Overall, 108 PET/CT reports, performed for the staging of NETs were analyzed: These were located in the pancreas (59.3%; *n* = 64), duodenum (14.8%; *n* = 16) and ileum (25.9%; *n* = 28). Figure 1A shows the workflow for LLM based prediction of TNM classification.

### UICC TNM classification

We evaluated the accuracy of predictions for primary tumor features (T), regional lymph node involvement (N) and distant metastasis (M) (Fig. 1B). ChatGPT-4o achieved microF1 scores of 0.84, 1.00 and 0.99 respectively; DeepSeek V3 scored 0.74, 1.00 and 0.99; Claude 3.5 Sonnet obtained 0.64, 0.89 and 0.99; and Gemini 2.0 Flash achieved 0.52, 0.97 and 0.99. Further details of the macro and micro F1 scores, recall, and precision for each attribute are summarized in Tables 1, 2, 3 and 4. The different models showed relatively similar performance in identifying the correct N and M stage, however, there were significant differences in calling the correct T stage. ChatGPT-4o scored 0.29 higher in T stage classification than Gemini 2.0 Flash (Fig. 1C).

**Table 1:** Overall performance of ChatGPT-4o on UICC TNM classification from PET/CT reports

ChatGPT-4o				
**Attribute**	**Precision**	**Recall**	**Macro F1**	**Micro F1**
T	0.84	0.83	0.71	0.79
N	0.98	0.99	0.99	0.99
M	0.99	0.98	0.99	0.99
Average	0.94	0.94	0.90	0.92

**Table 2:** Overall performance of deepseek V3 on UICC TNM classification from PET/CT reports

DeepSeek V3				
**Attribute**	**Precision**	**Recall**	**Macro F1**	**Micro F1**
T	0.65	0.68	0.55	0.65
N	0.99	0.99	0.99	0.99
M	0.99	0.99	0.99	0.99
Average	0.88	0.87	0.84	0.88

**Table 3:** Overall performance of Claude 3.5 sonnet on UICC TNM classification from PET/CT reports

Claude 3.5 Sonnet				
**Attribute**	**Precision**	**Recall**	**Macro F1**	**Micro F1**
T	0.59	0.6	0.48	0.58
N	0.95	0.99	0.97	0.97
M	0.98	0.99	0.99	0.99
Average	0.84	0.86	0.81	0.85

**Table 4:** Overall performance of gemini 2.0 flash on UICC TNM classification from PET/CT reports

Gemini 2.0 Flash				
**Attribute**	**Precision**	**Recall**	**Macro F1**	**Micro F1**
T	0.58	0.52	0.48	0.5
N	0.95	0.98	0.97	0.97
M	0.96	0.99	0.98	0.98
Average	0.83	0.83	0.81	0.82

#### Error analysis

**Fig. 2:**
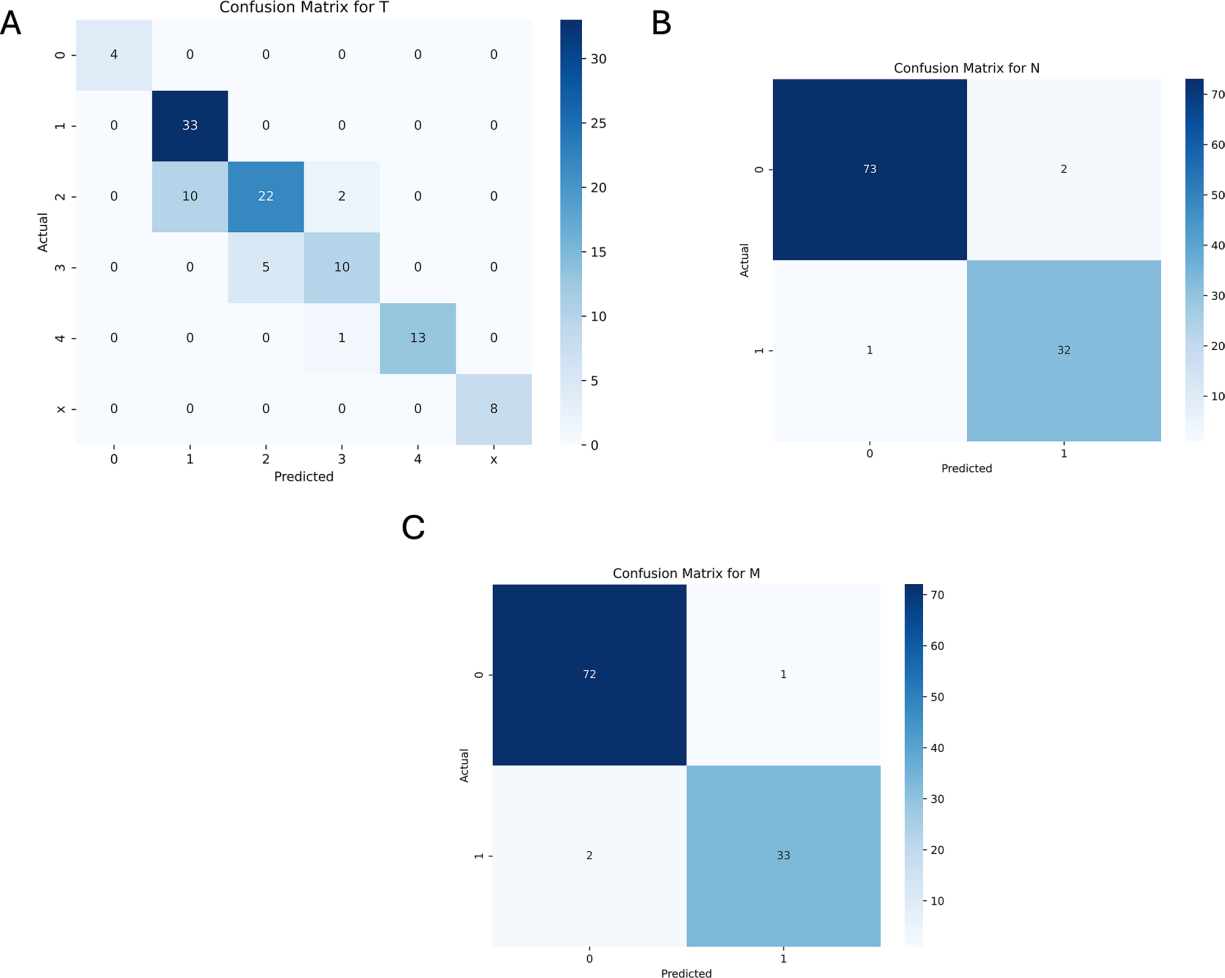
Confusion matrices for ChatGPT-4o for the key attributes of interest. (**A**) UICC T stage, (**B**) UICC N stage and (**C**) UICC M stage

To analyze the mistakes made by each model, we created confusion matrices for each model we used. For example, ChatGPT-4o (Fig. 2) confused the T2 stage for the T1 stage on 10 occasions, the M1 stage for M0 in two cases and N0 for N1 on two occasions. Confusion matrices for DeepSeek V3, Claude 3.5 Sonnet and Gemini 2.0 Flash can be seen in Suppl. Figure 1–3.

### ENETS TNM classification

**Fig. 3:**
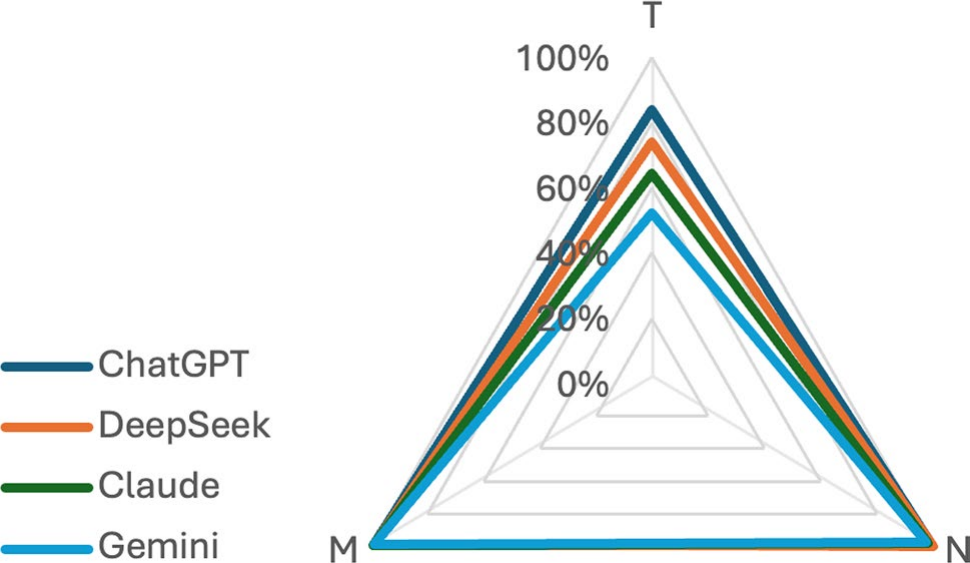
Radar chart illustrating the hit rate for ENETS TNM classification by GPT-4o, DeepSeek V3, Claude 3.5 Sonnet, and Gemini 2.0 Flash

We then used the four LLMs to classify the reports according to the ENETS classification system. The accuracy of primary tumor features (T), regional lymph node involvement (N), distant metastasis (M) was analyzed. ChatGPT-4o attained micro F1 scores of 0.84, 1.00, and 0.99, respectively. DeepSeek V3 recorded scores of 0.74, 1.00, and 0.99. Claude 3.5 Sonnet achieved 0.64, 0.89, and 0.99, while Gemini 2.0 Flash obtained scores of 0.52, 0.97, and 0.99. (Fig. 3)

Further details of the macro and micro F1 scores, recall, and precision for each attribute are summarized in Suppl. Tables 1–4.

#### Error analysis

**Fig. 4:**
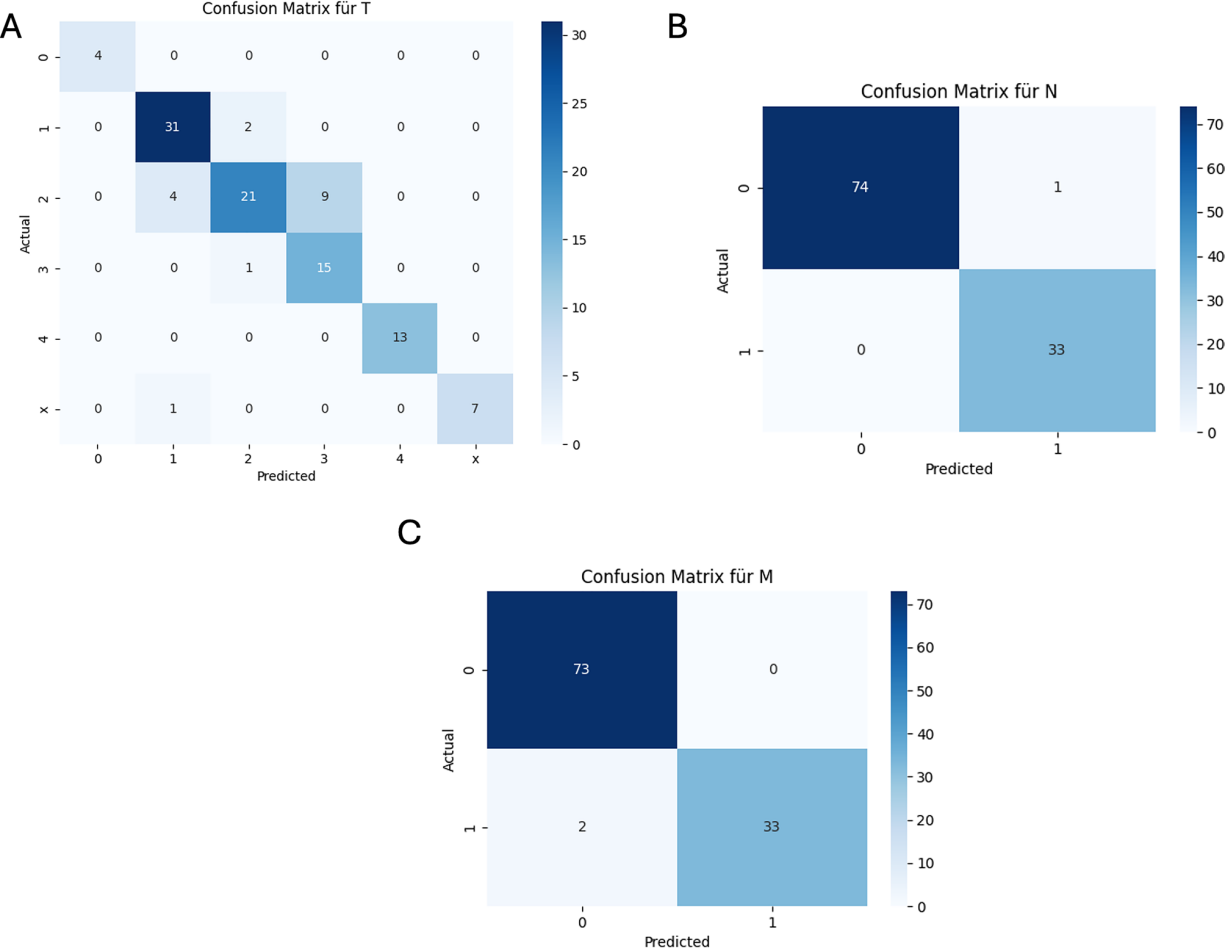
Confusion matrices for ChatGPT-4o for the key attributes of interest. (**A**) ENETS T stage, (**B**) ENETS N stage, (**C**) ENETS M stage

To evaluate the misclassifications made by each model for ENETS classification, we constructed confusion matrices for all tested models also for this classification. ChatGPT-4o mistakenly identified the T2 stage as T3 on nine occasions, confused M1 with M0 in two instances, and incorrectly labeled N0 as N1 once (Fig. 4). The confusion matrices for DeepSeek V3, Claude 3.5 Sonnet, and Gemini 2.0 Flash are available in the Supplementary Fig. 4–6.

## Discussion

This study highlights the transformative potential of large language models (LLMs) in the oncologic staging of neuroendocrine tumors (NETs), presenting an innovative solution to streamline complex diagnostic workflows. By accurately determining TNM staging from imaging reports, LLMs demonstrate their ability to interpret complex clinical narratives with high fidelity. Among the models evaluated, ChatGPT-4o achieved the highest performance, with microF1 scores of 0.79, 0.99 and 0.99, for T, N and M according to UICC and 0.84, 1.00 and 0.99 respectively, according to ENETS. These metrics underscore the model’s capability to support clinical workflows, potentially enhancing diagnostic efficiency and consistency. The comparatively lower performance observed for the T component likely reflects its dependence on nuanced anatomic details such as local invasion depth, size thresholds, and site-specific classification rules, which are often described less explicitly in PET/CT narratives than nodal or metastatic findings. Moreover, PET/CT inherently provides limited morphologic resolution for subtle features of local tumor extension, and variations in phrasing—such as “abuts,” “contacts,” or “suspicious for invasion”—can introduce additional ambiguity for language models. Future work could address these challenges by incorporating structured reporting elements, refining prompt design to capture context-specific descriptors, or combining text-based approaches with complementary imaging-derived information.

Past investigations into AI-powered medical text classification have predominantly focused on structured EHR data [27]. Previous research based on free-text has illustrated the potential of LLMs in a range of radiological classification applications. For example, one study [28] demonstrated that the LI-RADS score can be automatically derived from radiology reports, enhancing consistency in liver lesion evaluations. In another study [29], LLMs were employed to conduct TNM classification for NSCLC by extracting key tumor characteristics from free-text CT reports and translating them into standardized staging information. Moreover, LLMs have been effectively utilized in brain tumor classification [30], where they extracted and synthesized complex diagnostic details from radiology reports to support accurate clinical decision-making. In the current study, we expand upon this foundation by thoroughly examining cutting-edge LLMs in a particularly difficult diagnostic environment. Rather than using structured data, we drew on unstructured PET/CT reports, which are often laden with intricate, ambiguous phrasing, uncertain clinical terminology, and implied rather than explicitly stated diagnoses [31].

By contrast, no published research to date has addressed the ENET/UICC classification using LLMs exclusively on PET/CT reports.

A key insight from our analysis is the significant variation in performance across different LLMs. This variability highlights that while some models, like ChatGPT-4o, demonstrate superior performance, the specific factors contributing to this advantage remain unclear, and others may face challenges in effectively interpreting the nuanced nature of medical text. Therefore, understanding these discrepancies is crucial, underscoring the importance of rigorous model-specific evaluations and fine-tuning when considering LLMs for clinical applications.

The variability in reporting styles among physicians presents an additional challenge for LLM performance. Differences in terminology, structure, and the level of detail in imaging reports can significantly affect the models’ ability to accurately interpret and classify staging information. Some clinicians may prefer concise summaries, while others provide extensive descriptive narratives, which can introduce inconsistencies that LLMs must navigate. Addressing this challenge requires models to be trained on diverse datasets that encompass a wide range of reporting styles, ensuring greater robustness and adaptability in real-world clinical settings.

Another important consideration is the potential for LLMs to reduce variability in staging decisions among clinicians with differing levels of expertise. By providing consistent and reproducible assessments, LLMs could help standardize oncologic staging practices. This consistency could lead to more uniform treatment decisions, ultimately contributing to improved patient outcomes. Additionally, the ability of LLMs to rapidly process large volumes of imaging report data may alleviate the growing workload faced by healthcare professionals, allowing them to focus on more complex decision-making tasks that require human judgment.

Despite these encouraging results, several limitations should be acknowledged. First, this study relied on a physician-generated synthetic dataset of 108 PET/CT reports. While the use of fictitious cases ensured that no patient-identifiable information was included, the artificial nature of these reports may limit generalizability to real-world clinical practice, where reporting style, terminology, and level of detail can vary substantially. Nevertheless, the use of expertly designed synthetic reports offers distinct advantages: it enables controlled experimentation across predefined staging scenarios, ensures standardized wording and balanced representation of disease patterns, and eliminates privacy and ethical constraints associated with patient data. This controlled design allows systematic assessment of model behavior under defined conditions and represents an essential preparatory step toward prospective validation on authentic clinical reports. All analyses were descriptive in nature, and no formal hypothesis testing between models was performed. Finally, while no fabricated findings were observed in this study, large language models are known to be prone to hallucinations; this remains a potential risk—particularly with unconstrained prompts or real-world data—highlighting the need for appropriate safeguards and human oversight.

Our promising results on these standardized, yet variably phrased, synthetic reports lay the groundwork for future investigations with genuine patient data. Ultimately, confirming that these models perform similarly well on real-world datasets is critical for translation into routine clinical use.

## Conclusion

This study highlights the significant potential of LLMs, particularly GPT-4o, in automating the oncologic staging of NETs with high accuracy. The results suggest that LLMs can be valuable adjuncts in clinical decision-making, especially for non-specialists who may benefit from automated support in interpreting complex imaging reports. However, before LLMs can be integrated into routine clinical practice, prospective validation studies in diverse, real-world settings are essential. Additionally, addressing ethical, legal, and practical considerations will be key to their successful and responsible deployment. Ultimately, while LLMs hold great promise in enhancing oncologic workflows, their role should be viewed as complementary to, rather than a replacement for, expert clinical judgment.

## Supplementary Information

Below is the link to the electronic supplementary material.

Supplementary Material 1

Supplementary Material 2

## Data Availability

The datasets generated during and/or analysed during the current study are available from the corresponding author on reasonable request.
