# Supplementary material for: LLM-powered TNM staging of neuroendocrine tumors from PET/CT reports
Source: BMC Med Imaging. 2025 Dec 23;26:50. doi: 10.1186/s12880-025-02092-3 (PMC12838453; doi:10.1186/s12880-025-02092-3)
Supplement: Supplementary file 1 — Supplementary Material 1 [file 12880_2025_2092_MOESM1_ESM.pdf]

Supplementary Figures:

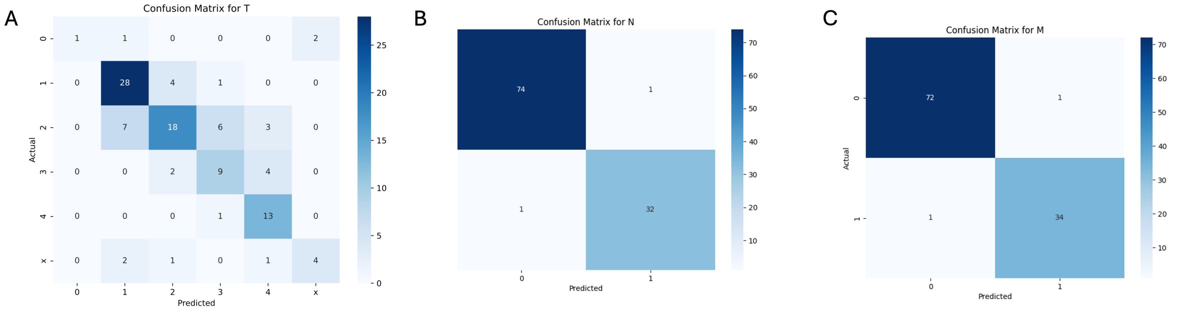

Suppl. Fig. 1: Confusion matrices for DeepSeek V3 for the key attributes of interest. (A) UICC T stage, (B) UICC N stage and (C) UICC M stage.

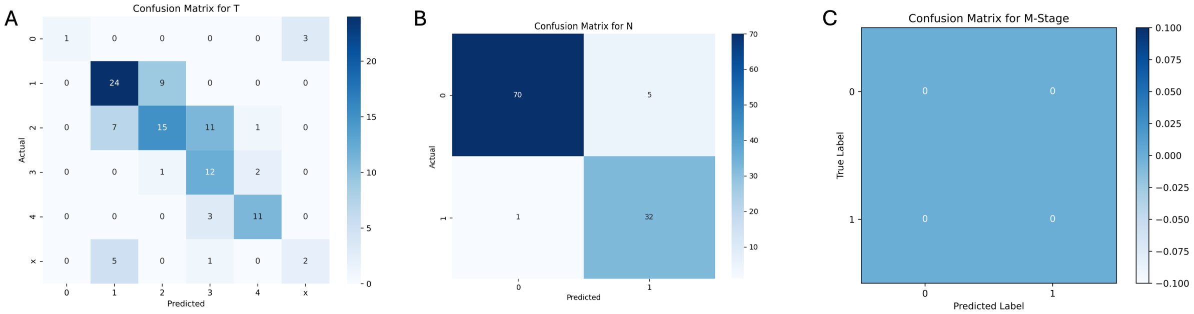

Suppl. Fig. 2: Confusion matrices for Claude 3.5 Sonnet for the key attributes of interest. (A) UICC T stage, (B) UICC N stage and (C) UICC M stage.

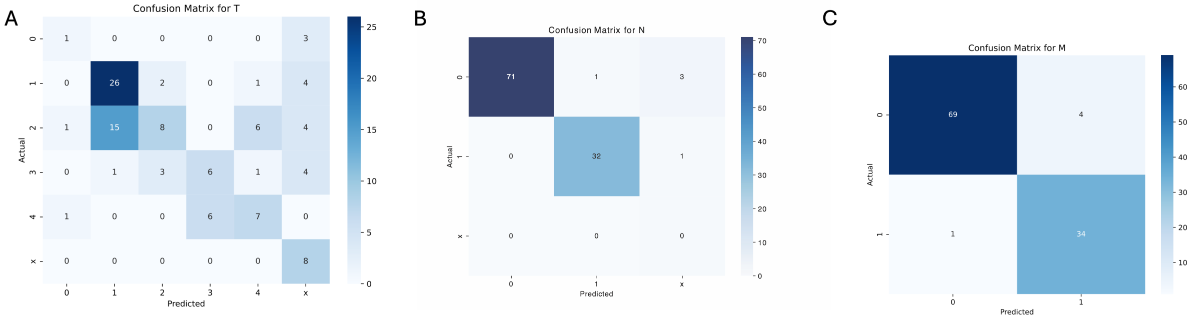

Suppl. Fig. 3: Confusion matrices for Gemini 2.0 Flash for the key attributes of interest. (A) UICC T stage, (B) UICC N stage and (C) UICC M stage.

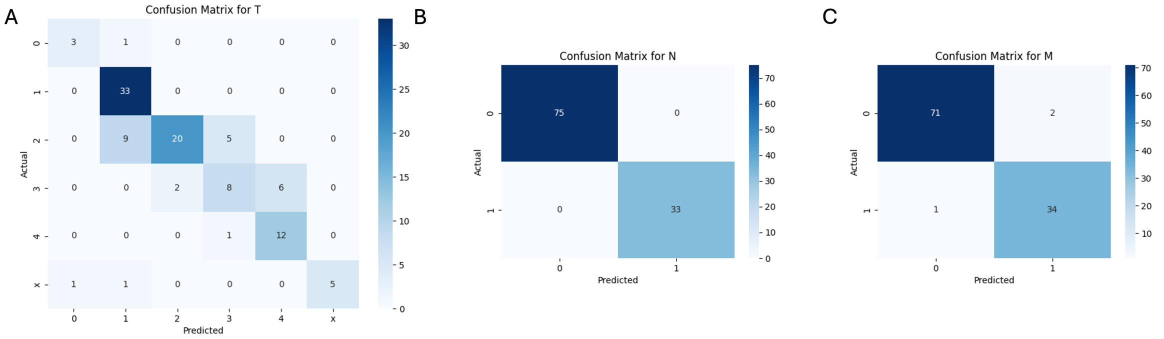

Suppl. Fig. 4: Confusion matrices for DeepSeekV3 for the key attributes of interest. (A) ENETS T stage, (B) ENETS N stage and (C) ENETS M stage.

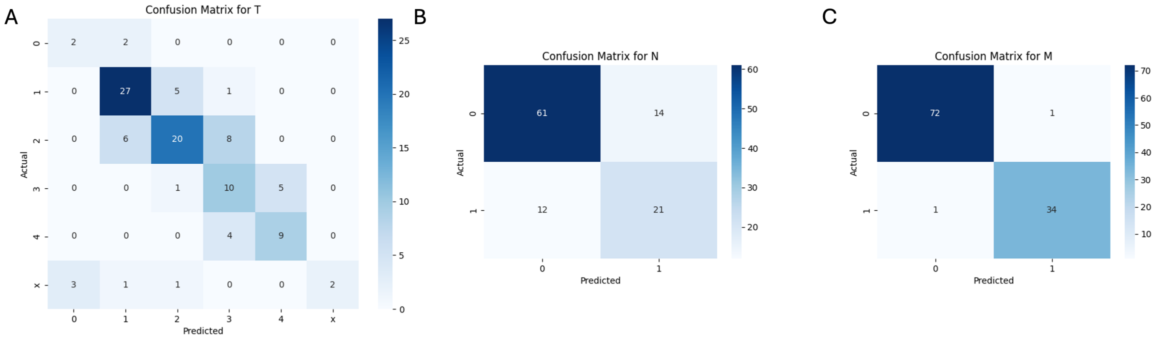

Suppl. Fig. 5: Confusion matrices for Claude 3.5 Sonnet for the key attributes of interest. (A) ENETS T stage, (B) ENETS N stage and (C) ENETS M stage.

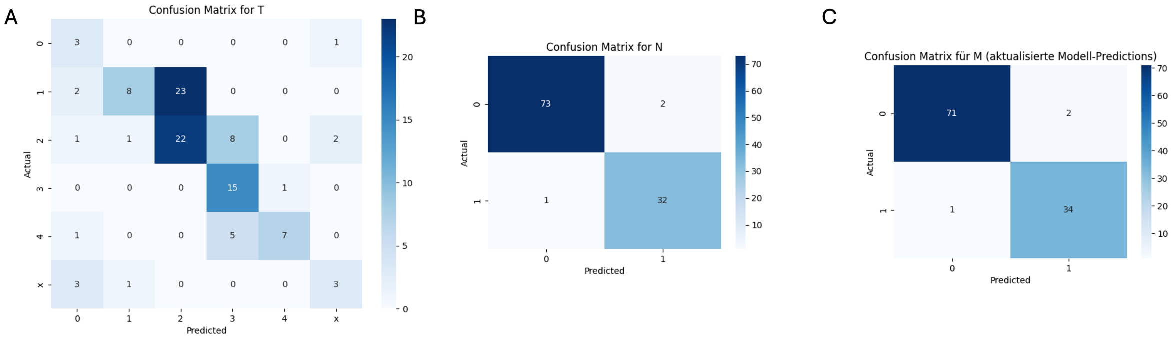

Suppl. Fig. 6: Confusion matrices for Gemini 2.0 Flash for the key attributes of interest. (A) ENETS T stage, (B) ENETS N stage and (C) ENETS M stage.

Supl. Tables:

ChatGPT-4o				
**Attribute**	**Precision**	**Recall**	**Makro F1**	**Micro F1**
T	0.84	0.83	0.71	0.79
N	0.98	0.99	0.99	0.99
M	0.99	0.98	0.99	0.99
Average	0.94	0.93	0.90	0.92

Table 1: Overall performance of ChatGPT-4o on ENETS TNM classification from PET/CT reports

DeepSeek V3				
**Attribute**	**Precision**	**Recall**	**Makro F1**	**Micro F1**
T	0.65	0.68	0.55	0.65
N	0.99	0.99	0.99	0.99
M	0.99	0.99	0.99	0.99
Average	0.88	0.89	0.84	0.88

Table 2: Overall performance of Claude 3.5 Sonnet on on ENETS TNM classification from PET/CT reports

Claude 3.5 Sonnet				
**Attribute**	**Precision**	**Recall**	**Makro F1**	**Micro F1**
T	0.59	0.6	0.48	0.58
N	0.95	0.99	0.97	0.97
M	0.98	0.99	0.99	0.99
Average	0.84	0.86	0.81	0.85

Table 3: Overall performance of Claude 3.5 Sonnet on ENETS TNM classification from PET/CT reports

Gemini 2.0 Flash				
**Attribute**	**Precision**	**Recall**	**Makro F1**	**Micro F1**
T	0.58	0.52	0.48	0.5
N	0.95	0.98	0.97	0.97
M	0.96	0.99	0.98	0.98
Average	0.83	0.83	0.81	0.82

Table 4: Overall performance of Gemini 2.0 Flash on ENETS TNM classification from PET/CT reports
