# Supplementary material for: LLM-powered TNM staging of neuroendocrine tumors from PET/CT reports
Source: BMC Med Imaging. 2025 Dec 23;26:50. doi: 10.1186/s12880-025-02092-3 (PMC12838453; doi:10.1186/s12880-025-02092-3)
Supplement: Supplementary file 2 — Supplementary Material 2 [file 12880_2025_2092_MOESM2_ESM.docx]

| Report | Groundtruth | GPT4o | DeepSeek | Claude | Gemini |
| --- | --- | --- | --- | --- | --- |
| Neck/Thorax: Moderately increased focal tracer uptake in the cranial upper pole of the left thyroid lobe (SUVmax 6.1), corresponding to a slightly hypodense lesion, most consistent with a nodular goiter. No lymph nodes or pulmonary lesions suspicious for malignancy.  Abdomen/Pelvis: Intensely tracer-avid, hyperdense tissue proliferation protruding convexly into the lumen of the terminal ileum (approximately 13 × 15 mm, SUVmax 43). Otherwise, no lymph nodes or lesions suspicious for malignancy in the upper abdominal or pelvic organs. Arterially hypervascularized area in liver segment 2 (8 mm) without increased tracer uptake, most consistent with a hemangioma. Mean hepatic SUV: 7.8.  Skeleton: No osseous lesions suspicious for malignancy.  Impression:Intensely somatostatin receptor–expressing tissue proliferation in the terminal ileum, well compatible with the histologically confirmed neuroendocrine tumor (NET). No evidence of metastases. | T2N0M0 | T2N0M0 | T2N0M0 | T2N0M0 | T2N0M0 |

*Supplementary Table 1. Example PET/CT report with corresponding ground-truth TNM classification and model-generated outputs.*
